# Epilepsy in *kcnj10* Morphant Zebrafish Assessed with a Novel Method for Long-Term EEG Recordings

**DOI:** 10.1371/journal.pone.0079765

**Published:** 2013-11-14

**Authors:** Anselm A. Zdebik, Fahad Mahmood, Horia C. Stanescu, Robert Kleta, Detlef Bockenhauer, Claire Russell

**Affiliations:** 1 Department of Neuroscience, Physiology and Pharmacology, University College London, London, United Kingdom; 2 Department of Comparative Biological Sciences, Royal Veterinary College, London, United Kingdom; 3 Centre for Nephrology, University College London, London, United Kingdom; 4 Institute of Child Health, University College London, London, United Kingdom; McGill University, Canada

## Abstract

We aimed to develop and validate a reliable method for stable long-term recordings of EEG activity in zebrafish, which is less prone to artifacts than current invasive techniques. EEG activity was recorded with a blunt electrolyte-filled glass pipette placed on the zebrafish head mimicking surface EEG technology in man. In addition, paralysis of agarose-embedded fish using D-tubocurarine excluded movement artifacts associated with epileptic activity. This non-invasive recording technique allowed recordings for up to one hour and produced less artifacts than impaling the zebrafish optic tectum with a patch pipette. Paralyzed fish survived, and normal heartbeat could be monitored for over 1h. Our technique allowed the demonstration of specific epileptic activity in *kcnj10a* morphant fish (a model for EAST syndrome) closely resembling epileptic activity induced by pentylenetetrazol. This new method documented that seizures in the zebrafish EAST model were ameliorated by pentobarbitone, but not diazepam, validating its usefulness. In conclusion, non-invasive recordings in paralyzed EAST syndrome zebrafish proved stable, reliable and robust, showing qualitatively similar frequency spectra to those obtained from pentylenetetrazol-treated fish. This technique may prove particularly useful in zebrafish epilepsy models that show infrequent or conditional seizure activity.

## Introduction

Most forms of epilepsy lack specific treatment. Recently, we elucidated the pathophysiological basis of a multisystem disorder characterized by early childhood epilepsy, ataxia, sensorineural deafness and a salt-wasting tubulopathy (EAST syndrome [1]). KCNJ10, expressed in glial cells, helps to buffer extracellular potassium and thus modulates neuronal excitability [2], [3] explaining epilepsy in this autosomal recessive disorder. Current treatment for this disorder caused by malfunction of the potassium channel KCNJ10 in affected organs is non-specific and unsatisfactory.

Zebrafish (ZF) can be maintained at low cost, and large numbers of embryos and larvae can be exposed to potential therapeutics simultaneously. They are thus ideally suited for *in vivo* screening [4]. Indeed, chemical screens to identify potential anticonvulsants have been performed [5]. We recently showed [6] that *kcnj10a* morphant larval ZF are a faithful model for EAST syndrome. These fish recapitulate key features of EAST syndrome including ataxia and a renal excretion defect. We have now developed a novel electroencephalographic method to assess their epileptic phenotype in stable recordings for up to one hour. Our method virtually eliminates artifacts caused by movement in seizing fish, and artifacts related to trauma caused by impalement, and therefore accurately reflects the EEG, i.e. field electrical activity in the zebrafish optic tectum.

## Materials and Methods

### Zebrafish lines and husbandry

Embryos were obtained by natural spawning from WT ZF (TupLongfin). All ZF were reared at 28°C according to standard procedures.

### Generation of seizure models

Morpholino oligonucleotides (Gene Tools, USA) targeting the start ATG (agggataggagagagatgttcattt) or a splice-site (aattgtgagagctataccttggcga) of ZF *kncj10a,* were diluted into morpholino buffer containing (in mM) 58 NaCl, 0.7 KCl, 0.4 MgSO_4_, 0.6 Ca(NO_3_)_2_, 5 HEPES-NaOH, pH 7.6, and injected into 1-2 cell stage ZF. For details see [6].

120 hours post fertilization (hpf) wildtype ZF were treated with 15 mM pentylenetetrazole (PTZ) in aquarium water (AW) for 2-10 min until twitching indicated seizure activity.

### EEG

**Figure 1:**
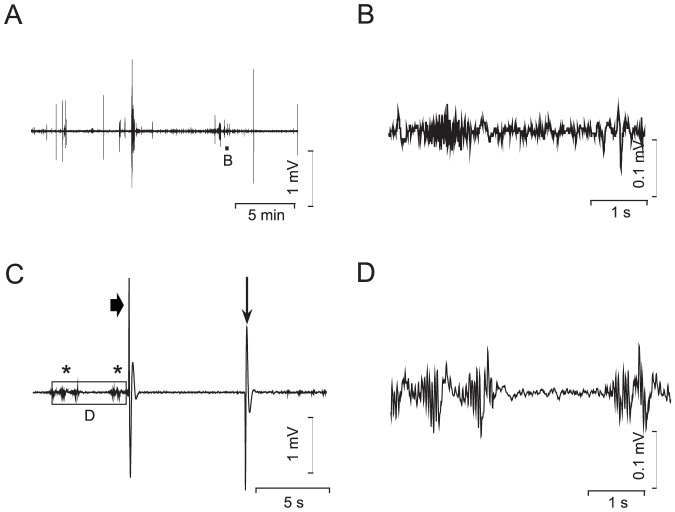
Invasive recordings using a patch pipette inserted into the optic tectum of 120 hpf old ZF larvae. (A), 22 min recording of a buffer-injected control larva. Frequent spiking and also low level activity is present throughout the trace. (B), higher temporal and voltage resolution of the area marked in A. (C), kcnj10a morphant larva, showing genuine epileptic activity (marked with stars). Low-level activity is followed by a spontaneous large transient (arrowhead). Similar transients were seen while the fish showed brief total body contractions and could also be produced by a light tap on the recording setup (vertical arrow). The square marked “D” in C is shown in (D) at higher resolution.

Initially, we inserted a patch pipette into the optic tectum, as described [7], [8]. However, in our hands fish movement and/or electrode placement in the brain could induce seizure-like electrical activity even in control fish (Fig. 1*A,B*). In order to avoid these artifacts, we developed a new method where surface recordings from the skin above the optic tectum and paralysis with D-tubocurarine reliably and completely abolished these artifacts. 120 hpf wild-type, *kcnj10a* morphant or PTZ-treated ZF were placed in 2 mM D-tubocurarine (Fluka, UK) in AW for 10 minutes, rinsed and mounted close to the surface in 1.5% Type VII low melting point agarose (Sigma, UK) in AW. Recording electrodes were pulled from borosilicate glass with filament (GC150 TF- 7.5, Harvard Apparatus, UK) on a Zeitz Universalpuller (Zeitz, Germany), broken to a tip diameter of 10–15 µm, fire polished and filled with 1 M NaCl. The field potential between the recording electrode placed on the skin and a reference electrode placed into the agarose was amplified 10,000x using a DAGAN™ 2400 amplifier (Minnesota, USA), band pass filtered at 0.3–300 Hz and digitized at 2 kHz via a PCI-6251 interface (National Instruments, UK) using WinEDR (John Dempster, University of Strathclyde, UK). ZF were recorded for up to one hour whilst heartbeat and peripheral circulation were good, as monitored by microscopic inspection. Fourier analysis was performed in Origin on representative 20 s stretches and data were averaged over all experiments and over the 2–4 Hz band for statistical analysis.

### Antiepileptic drugs

Diazepam (Sigma, UK) was predissolved in DMSO (Fluka, UK) at 100 mM and diluted to 1 mM in AW. Pentobarbitone-Na solution (Pentoject, Animalcare Ltd. UK) was diluted 1∶10 in AW to 20 mg/ml. Drug effects were observed approximately 15 min after addition to the AW surrounding the agarose-embedded fish.

### Statistical analysis

Statistical analysis was performed in Origin (OriginLab, USA) with the two-sided unpaired or paired Student’s t-test. p<0.05 was considered significant.

### Animal experiments

All ZF experiments were approved by Royal Veterinary College, UCL and the UK Home Office.

## Results

### Knock-down of ZF *kcnj10a*

Antisense morpholino oligonucleotides (MO) were designed against a donor splice site (intron 2) and the start codon of *kcnj10a,* as described [6]. Fish injected with up to 2 ng of either MO displayed no gross dysmorphology, but showed spontaneous contractions at 30 hpf (hours post fertilization), consistent with epileptic seizures. This “twitching” reverted to normal levels when human WT cRNA was co-injected, but not *KCNJ10* R65P [6], a missense mutation causing EAST syndrome in humans [1], [9]–[12].

### Seizures in pentylenetetrazole-treated and *kcnj10a* morphant zebrafish

**Figure 2:**
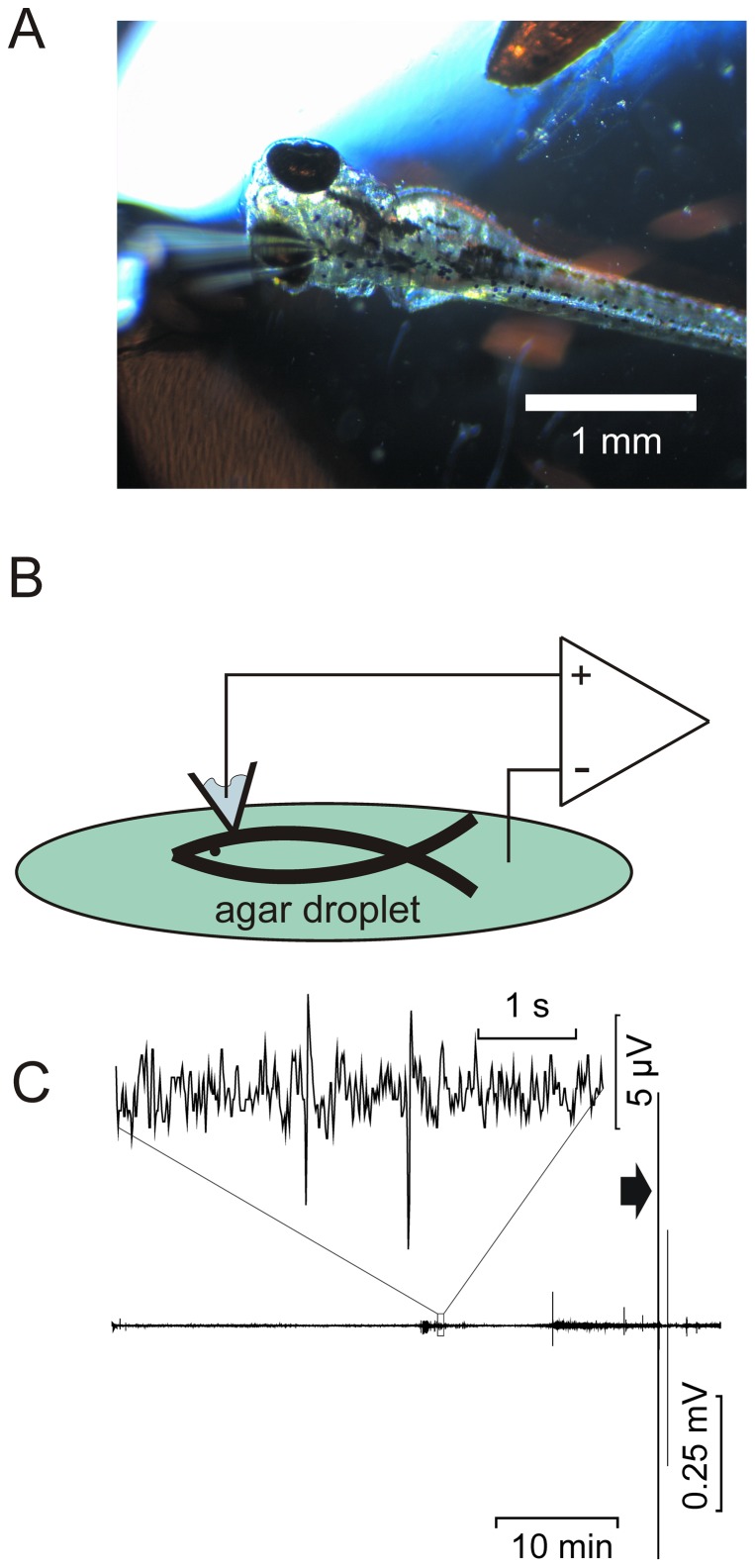
Non-invasive recordings using a patch pipette on the surface of the optic tectum. (A) Photo of the non-invasive EEG ZF recording set-up. To the left is a surface recording pipette, filled with 1 M NaCl. In the upper right is the reference electrode. Both are connected to the amplifier, as shown in the schematic in (B). The mounted fish is positioned on a microscope with which ZF viability can be monitored continuously. (C), long-term EEG recording of a kcnj10a morphant fish initially paralyzed in 20 mM D-tubocurarine. Fish were generally viable for over an hour, but paralysis appeared to wear off after 50 to 60 min. Movement artifacts ensued, associated with electrical activity and visible twitching (arrowhead). A 5 s period indicated by a box in C is represented above the trace in higher resolution.

**Figure 3:**
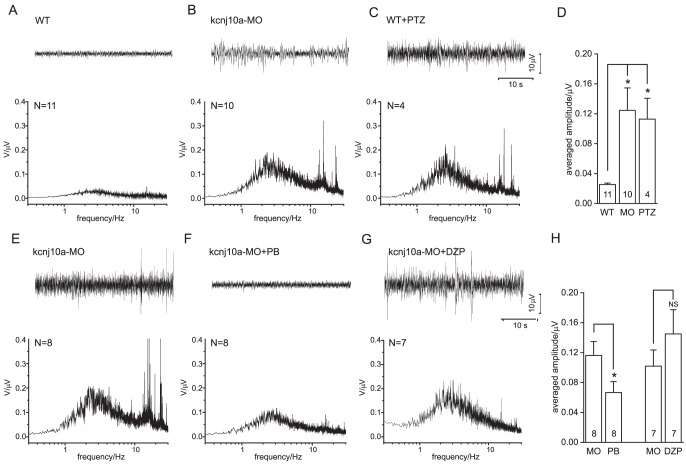
Analysis of surface EEGs. (A-C) Original traces (top) and averaged frequency spectra (below) obtained in WT ZF, kcnj10a morphant ZF and WT ZF pretreated with pentylenetetrazol. (D) The averaged amplitudes (mean over 2–4 Hz) from (A-C) are compared. Note the significant epileptic activity in kcnj10a morphant (MO) and pentylenetetrazol-treated (PTZ) ZF. (E-G) Original traces (top) and averaged frequency spectra (below) obtained in kcnj10a morphant fish, and kcnj10a morphants treated with pentobarbitone (PB) or diazepam (DZP), respectively. (H) Averaged amplitudes (mean over 2–4 Hz) from (E-G) are compared. Note the significant suppression of epileptic activity by PB treatment, and lack of suppression by DZP.

At 120 hpf we frequently observed a rapid increase in locomotion accompanied by a reduced ability to change direction, followed by a loss-of-posture in *kcnj10a* morphant ZF [6], as observed in PTZ-induced ZF models of epilepsy and the *mind bomb* mutant [8], [13]. We therefore asked whether movement abnormalities in morphant fish reflected seizures, and initially performed electroencephalogram (EEG) recordings at 120 hpf with a patch pipette inserted into the optic tectum as described [8] (Fig. 1). Insertion of the patch electrode through the skin often significantly blunted these pipettes to >10 µm tip diameter (data not shown). In addition, trauma, presumably caused by excessive leak of pipette solution into the optic tectum, frequently led to tissue opacity in the vicinity of the electrode (data not shown). More importantly, we frequently recorded artifacts with this technique in control-injected larvae (Fig. 1 *A,B*), including movement artifacts indistinguishable from seizure activity, which also could be mimicked by light tapping on the table carrying the recording setup, as shown in Fig. 1. Therefore, signals recorded with this technique from twitching fish very likely reflected both electrical seizure activity as well as movement artifacts. Insertion of a patch pipette into the zebrafish brain, i.e., the optic tectum, was also rarely tolerated for more than 15 min, making recording of the intermittent epileptic activity, which we frequently observed in *kcnj10a* morphant fish, difficult. We therefore developed a technique imitating a surface EEG as obtained from humans in clinical practice by placing a single glass electrode on the skin overlying the optic tectum (Fig. 2*A*). This non-invasive method allowed for more stable long-term recordings and proved much less sensitive to vibration, and was therefore employed for all subsequent recordings shown here. To further prevent artifacts we limited larval movement. ZF were paralyzed with D-tubocurarine prior to immobilization in agarose. When assessed with this new technique, recordings were stable for approximately 50 min (Fig. 2*B*). Note that synchronized activity in this ZF morpholino model is only apparent after 25 min (box in C enlarged above the trace), necessitating stable long-term recordings. In order to verify that our technique was sensitive enough to pick up genuine epileptic activity, we also recorded from fish treated with the pro-convulsant pentylenetetrazole [8] (Fig. 3*A-C*). These fish exhibited electrical spikes in clusters of variable length, occasionally transforming into continuous spiking (Fig. 3*C*), which was followed by death. The *kcnj10a* morphant fish showed similar activity, albeit less pronounced, and there were long periods of relative silence in the field electrical activity (Fig. 2*C*). Fourier analysis on representative stretches showed increased power in the 2–4 Hz frequency band for both *kcnj10a* MO and pentylenetetrazole-treated fish, a frequency band typical also for human seizure activity. For statistical evaluation, we averaged the spectra over 2–4 Hz and all experiments (Fig. 3*D*).

### Antiepileptic treatment suppresses synchronized brain activity in *kcnj10a* morphant fish

We applied pentobarbitone (PB) and diazepam (DZP) to 120 hpf *kcnj10a* morphant ZF. Whereas PB effectively suppressed the dominating seizure activity (Fig. 3*F*), no such effect was seen after administration of DZP (Fig. 3*G*). The PB effect was statistically significant when comparing the averaged EEG spectra (Fig. 3*H*). Interestingly, DZP suppressed higher frequencies ∼15 Hz only.

## Discussion and Conclusion

ZF provide an excellent model for the study of human disease. Knock out mice for KCNJ10 show an early lethal phenotype making this model less suitable for epilepsy research [14]. In contrast a morpholino knock down of Kcnj10a in ZF generates a phenotype mimicking EAST syndrome [6] and therefore ZF are well suited to study its neurology [1].

Further characterization of the epileptic phenotype in EAST syndrome required refinement of the published technique [8] for recording seizure activity in ZF larvae, as it was prone to artifacts. It also rarely allowed long recordings, which would exclude drug testing on the same fish. We therefore sought a noninvasive and more robust technique. EEGs have been recorded in humans with surface electrodes for decades and provide a robust electrical signal. Whereas this signal is generated by a cortex at least 100 times larger than the optic tectum of ZF, the smaller anatomy and better conductivity of the skin in ZF larvae should actually provide better recording conditions. Indeed, a surface EEG recorded over the optic tectum using a blunt, fire-polished pipette allowed us to record robust, albeit smaller amplitude signals for up to 1 h. It was also less sensitive to vibration than the previously published technique [8] but more alike a technique published for adult ZF [15]. We further reduced potential movement artifacts by paralyzing fish with D-tubocurarine prior to embedding in low melting point agarose.

Fourier analysis has not previously been applied widely to ZF epilepsy data. We performed Fourier analysis on the recordings obtained from *kcnj10a* morphant fish with our improved method and found increased power in the 2–4 Hz band, resembling some forms of epilepsy in humans [16], [17]. Epilepsy is a common disorder in humans as it affects as much as 0.5% of the population over their lifetime, yet is poorly understood. Consequently, pharmacological treatment of epilepsy remains largely empirical. Yet, genetic variants in KCNJ10 have been associated with idiopathic epilepsy [18]–[20]. We therefore endeavoured to test our method in characterising the action of antiepileptic drugs in our ZF EAST model. The differential effect of PB and DZP on electrical activity over the brain of *kcnj10a* morphant fish provides proof-of-principle that our model can be used to identify novel or improved therapeutic substances. We speculate that DZPs selective action on GABA-A receptor channels is less effective on epilepsy resulting from elevated extracellular potassium [3]. As we can essentially “humanize” this model (rescuing the morphant phenotype by coinjection of human WT but not mutant cRNA) [6], mutation-specific drug testing is possible.

In summary we have developed a new EEG technique for recording synchronized activity in ZF and show that this technique improves data acquisition from this emerging model organism. We demonstrate that it records chemically induced epileptic activity, as well as epileptic activity in a defined genetic epilepsy model of EAST syndrome, and that this model is well suited for screening pharmacological agents.

Our ZF epilepsy model is a replacement model and could also help to reduce the number of rodents used in anticonvulsant screening programs dramatically.

## References

[1] Bockenhauer D, Feather S, Stanescu HC, Bandulik S, Zdebik AA, et al (2009) Epilepsy, ataxia, sensorineural deafness, tubulopathy, and KCNJ10 mutations. N Engl J Med 360: 1960–1970. 19420365 10.1056/NEJMoa0810276 PMC3398803

[2] Olsen ML, Sontheimer H (2008) Functional implications for Kir4.1 channels in glial biology: from K+ buffering to cell differentiation. Journal of neurochemistry 107: 589–601. 18691387 10.1111/j.1471-4159.2008.05615.x PMC2581639

[3] Haj-Yasein NN, Jensen V, Vindedal GF, Gundersen GA, Klungland A, et al (2011) Evidence that compromised K+ spatial buffering contributes to the epileptogenic effect of mutations in the human Kir4.1 gene (KCNJ10). Glia 59: 1635–1642. 21748805 10.1002/glia.21205

[4] Santoriello C, Zon LI (2012) Hooked! Modeling human disease in zebrafish. J Clin Invest 122: 2337–2343. 22751109 10.1172/JCI60434 PMC3386812

[5] Baxendale S, Holdsworth CJ, Meza Santoscoy PL, Harrison MR, Fox J, et al (2012) Identification of compounds with anti-convulsant properties in a zebrafish model of epileptic seizures. Dis Model Mech 5: 773–784. 22730455 10.1242/dmm.010090 PMC3484860

[6] Mahmood F, Mozere M, Zdebik AA, Stanescu HC, Tobin J, et al (2013) Generation and validation of a zebrafish model of EAST (epilepsy, ataxia, sensorineural deafness and tubulopathy) syndrome. Dis Model Mech 6: 652–60. 23471908 10.1242/dmm.009480 PMC3634649

[7] Baraban SC, Dinday MT, Castro PA, Chege S, Guyenet S, et al (2007) A large-scale mutagenesis screen to identify seizure-resistant zebrafish. Epilepsia 48: 1151–1157. 17521353 10.1111/j.1528-1167.2007.01075.x PMC2211740

[8] Baraban SC, Taylor MR, Castro PA, Baier H (2005) Pentylenetetrazole induced changes in zebrafish behavior, neural activity and c-fos expression. Neuroscience 131: 759–768. 15730879 10.1016/j.neuroscience.2004.11.031

[9] Bandulik S, Schmidt K, Bockenhauer D, Zdebik AA, Humberg E, et al (2011) The salt-wasting phenotype of EAST syndrome, a disease with multifaceted symptoms linked to the KCNJ10 K+ channel. Pflugers Archiv : European journal of physiology 461: 423–435. 21221631 10.1007/s00424-010-0915-0

[10] Freudenthal B, Kulaveerasingam D, Lingappa L, Shah MA, Brueton L, et al (2011) KCNJ10 Mutations Disrupt Function in Patients with EAST Syndrome. Nephron Physiol 119: p40–p48. 21849804 10.1159/000330250

[11] Reichold M, Zdebik AA, Lieberer E, Rapedius M, Schmidt K, et al (2010) KCNJ10 gene mutations causing EAST syndrome (epilepsy, ataxia, sensorineural deafness, and tubulopathy) disrupt channel function. Proc Natl Acad Sci U S A 107: 14490–14495. 20651251 10.1073/pnas.1003072107 PMC2922599

[12] Thompson DA, Feather S, Stanescu HC, Freudenthal B, Zdebik AA, et al (2011) Altered electroretinograms in patients with KCNJ10 mutations and EAST syndrome. J Physiol 589: 1681–1689. 21300747 10.1113/jphysiol.2010.198531 PMC3099023

[13] Hortopan GA, Dinday MT, Baraban SC (2010) Spontaneous seizures and altered gene expression in GABA signaling pathways in a mind bomb mutant zebrafish. J Neurosci 30: 13718–13728. 20943912 10.1523/JNEUROSCI.1887-10.2010 PMC2962868

[14] Neusch C, Papadopoulos N, Muller M, Maletzki I, Winter SM, et al (2006) Lack of the Kir4.1 channel subunit abolishes K+ buffering properties of astrocytes in the ventral respiratory group: impact on extracellular K+ regulation. Journal of neurophysiology 95: 1843–1852. 16306174 10.1152/jn.00996.2005

[15] Pineda R, Beattie CE, Hall CW (2011) Recording the adult zebrafish cerebral field potential during pentylenetetrazole seizures. J Neurosci Methods 200: 20–28. 21689682 10.1016/j.jneumeth.2011.06.001 PMC5503205

[16] Bauzano-Poley E, Rodriguez-Barrionuevo AC (2001) [Electroencephalographic diagnosis of the idiopathic generalized epilepsies of childhood]. Rev Neurol 32: 365–372. 11333394

[17] Westmoreland BF (1996) Epileptiform electroencephalographic patterns. Mayo Clin Proc 71: 501–511. 8628033 10.4065/71.5.501

[18] Buono RJ, Lohoff FW, Sander T, Sperling MR, O'Connor MJ, et al (2004) Association between variation in the human KCNJ10 potassium ion channel gene and seizure susceptibility. Epilepsy Res 58: 175–183. 15120748 10.1016/j.eplepsyres.2004.02.003

[19] Ferraro TN, Golden GT, Smith GG, Martin JF, Lohoff FW, et al (2004) Fine mapping of a seizure susceptibility locus on mouse Chromosome 1: nomination of Kcnj10 as a causative gene. Mammalian genome 15: 239–251. 15112102 10.1007/s00335-003-2270-3

[20] Lenzen KP, Heils A, Lorenz S, Hempelmann A, Hofels S, et al (2005) Supportive evidence for an allelic association of the human KCNJ10 potassium channel gene with idiopathic generalized epilepsy. Epilepsy Res 63: 113–118. 15725393 10.1016/j.eplepsyres.2005.01.002

